# Qualitative Environmental Health Research: An Analysis of the Literature, 1991–2008

**DOI:** 10.1289/ehp.0901762

**Published:** 2010-04-26

**Authors:** Madeleine Kangsen Scammell

**Affiliations:** Department of Environmental Health, Boston University School of Public Health, Boston, Massachusetts, USA

**Keywords:** anthropology, environmental epidemiology, mixed methods, participatory research, qualitative methods, sociology, theory

## Abstract

**Background:**

Recent articles have advocated for the use of qualitative methods in environmental health research. Qualitative research uses nonnumeric data to understand people’s opinions, motives, understanding, and beliefs about events or phenomena.

**Objective:**

In this analysis of the literature, I report the use of qualitative methods and data in the study of the relationship between environmental exposures and human health.

**Data sources:**

A primary search on ISI Web of Knowledge/Web of Science for peer-reviewed journal articles dated from 1991 through 2008 included the following three terms: qualitative, environ*, and health. Inclusion and exclusion criteria are described.

**Data extraction:**

Searches resulted in 3,155 records. Data were extracted and findings of articles analyzed to determine where and by whom qualitative environmental health research is conducted and published, the types of methods and analyses used in qualitative studies of environmental health, and the types of information qualitative data contribute to environmental health.

**Data synthesis:**

Ninety-one articles met inclusion criteria. These articles were published in 58 different journals, with a maximum of eight for a single journal. The results highlight a diversity of disciplines and techniques among researchers who used qualitative methods to study environmental health, with most studies relying on one-on-one interviews. Details of the analyses were absent from a large number of studies. Nearly all of the studies identified increased scientific understanding of lay perceptions of environmental health exposures.

**Discussion and conclusions:**

Qualitative data are published in traditionally quantitative environmental health studies to a limited extent. However, this analysis demonstrates the potential of qualitative data to improve understanding of complex exposure pathways, including the influence of social factors on environmental health, and health outcomes.

Qualitative methods are commonly used in the social sciences and in a variety of disciplines related to public health. Several articles published as editorials or commentaries in public and environmental health journals in recent years have advocated for the use of more qualitative methods in environmental health research (Brown 2003; Foster and Sharp 2005; Lobdell et al. 2005; Rice et al. 2003).

Qualitative research frequently refers to a variety of approaches and techniques that may vary depending on the discipline (Snape and Spencer 2003). What they share is the recognition that when studying the social world, methodology must allow for the analysis of the construction of socially and culturally derived meaning and of human interpretation of reality. Qualitative studies are generally designed to explore perceptions of reality or, more specifically, perceptions of a phenomenon.

There are a number of widely agreed upon characteristics of qualitative research. Sources of qualitative data can be grouped into three categories: interviews (one-on-one and group), observations, and documents (Patton 2002). Qualitative interviews are often designed to ask open-ended questions, enabling the researcher to hear and make sense of responses from the people who are being interviewed without predetermining their points of view by fixing response categories ahead of time, as in quantitative survey methodology. Conventional survey methods do not allow for additional, surprising, or multifaceted responses. Questions likely to elicit a “yes” or “no” response are not open-ended, nor are questions that lead people to a type of response, for example, not stressful, somewhat stressful, very stressful. Another characteristic of qualitative research is the explicit consideration of the researchers’ perspective. Qualitative researchers are usually a primary data collection instrument (i.e., as opposed to a written survey instrument or an air monitor). Explicit recognition of theoretical perspectives helps researchers check and control potential biases in data interpretation. Reflexivity and bracketing are both forms of self-reflection practiced by qualitative researchers and involve evaluation of their roles in unintentionally tainting or manipulating data (Finlay 2002; Patton 2002).

One important aim of qualitative analysis is to make sense of the data while allowing the voice of the participants to be heard. A common way to do this is by using quotes and narrative descriptions in the output of qualitative research. Finally, analysis of qualitative data requires some degree of abstraction or generalization as patterns are identified in the data and related to larger constructs or theories. A definition of theory frequently cited by qualitative scholars is “a set of interrelated constructs, definitions, and propositions that present a rational view of phenomena by explaining or predicting relationships among those elements” (Ulin et al. 2005). The theoretical contributions of qualitative research come from the interpretation and analysis of qualitative data.

As for the theoretical relationship between qualitative data and environmental health, two overarching paradigms—interpretivism and positivism—characterize how people view the world and, some would argue, imply how the world should be studied (Ulin et al. 2005). (Within each are various nuanced theoretical traditions.) Positivist methods are based on the belief that the world consists of observable facts that exist, or are true, independent of human cognition (Wing 2003). Usually such observations are quantitative measurements (i.e., enumeration of the “independent variables” and their relationship with the “dependent variables”) (Wing 1994). On the other hand, interpretivism is concerned with the *meaning* of reality, not with measuring reality per se. Some interpretivist scholars would assert that no single reality exists and that all reality is filtered through the perception of human cognition (Ulin et al. 2005). According to this logic, because all observations are acts of unconscious interpretation, interpretivist research focuses on meanings and is usually represented by qualitative assessments (Ulin et al. 2005). In February 2005, a commentary (Foster and Sharp 2005) published in *Environmental Health Perspectives* (*EHP*) suggested the use of qualitative data as a means for generating hypotheses as well as for facilitating the multilevel analysis of individual, contextual, and structural factors that contribute to complex diseases. The authors, an anthropologist and a philosopher, recommended that researchers consider a hybrid study design that includes qualitative and quantitative methods. For example, when studying disease susceptibility, Foster and Sharp (2005) suggested that qualitative data will “empirically specify quantitatively testable practices rather than proxy identities or categories such as culture, ethnicity, gender, and class.” They concluded that “qualitative methods such as ethnography may become an interdisciplinary companion to epidemiology.” Rice et al. (2003) also suggested that qualitative data may help explain variation in quantitative exposure methods.

Three months after the Foster and Sharp article appeared, a feature article of the *Journal of Environmental Health* (Lobdell et al. 2005), whose first author is an epidemiologist at the U.S. Environmental Protection Agency, argued that qualitative research methods are underused by environmental health researchers. Lobdell et al. (2005) suggested a variety of ways that focus groups are and may be used to study environmental health, described specific techniques for conducting focus groups, and provided how-to references for interested readers.

The appreciation for qualitative methods by epidemiologists is not new. According to Dunn and Janes (1986), qualitative anthropological knowledge has been considered “useful” by epidemiologists since the 1950s with many collaborative studies conducted through the 1970s. Interdisciplinary research involving anthropologists and epidemiologists was most common in studies conducted in non-Western societies and among migrant groups in the United States and Europe. The focus of these studies was usually behavior and its role in disease etiology, which led to the development of strategies for modifying behavior (Trostle 1986b). Trostle (1986a) observed that when qualitative data are included in epidemiological studies, anthropologists would often “find themselves working primarily as epidemiologists,” as opposed to what is more recently referred to as transdisciplinary research where methods transcend the techniques of any single discipline (Rosenfield 1992; Stokols 2006). Trostle (1986a) cited few instances where anthropologists and epidemiologists worked as coarchitects in the creation of hybrid or new study designs.

Recent articles on the value of qualitative data in the study of environmental health argue that qualitative methods are especially important to community-based environmental health research because of their ability to engage residents regarding local environmental health problems. Qualitative methods “provide a way to produce community narratives that give voice to individuals and characterize the community in a full and complex fashion” (Brown 2003).

Such editorials and commentaries are compelling, but with few exceptions they do not include evidence of environmental epidemiologists using qualitative data or working with qualitative social scientists to study the relationship between environmental exposures and health outcomes. This article presents the results of an analysis of qualitative methods and data used in the study of environmental health and published in peer-reviewed scientific journals. The studies included in this analysis are ones that used nonnumerical, qualitative data on the relationship between environmental exposures and human health.

The objectives of this study were to identify where and by whom qualitative environmental health research is conducted and published, examine the types of methods and analyses used in qualitative studies of environmental health, and determine what types of information qualitative data contribute to the study of environmental health.

## Data Sources

Here, I review environmental health research that was published as a journal article, used qualitative methods, and was published in English. Books and monographs are not included. In the natural sciences, published articles are “the coin of the realm,” for promotion and recognition, rather than the book-length format more common in the humanities and social sciences. Each form of discourse has a value specific to its context. This analysis is limited to the type of discourse represented in peer-reviewed journal articles.

### Inclusion criteria

All studies included in this analysis report findings from qualitative research. Mixed-method studies that use both qualitative and quantitative techniques to collect and analyze data are also included. Although environmental health has typically concerned itself with the physical human health outcomes of exposure to environmental hazards, this analysis expands the definition of health effect to include mental and psychosocial health outcomes. Several articles are included that examined exercise or physical activity as an outcome.

Exposures considered in this analysis include physical, chemical, and biological exposures in people’s immediate or proximate surroundings (e.g., soil, air, water, food, and homes) and that affect people in their neighborhoods, communities, or workplaces. Articles that focus on social determinants of health as an exposure are included in this analysis only when examined in relation to a specific chemical, physical, or biological environmental exposure.

### Exclusion criteria

A large number of qualitative articles with a focus on the transmission of biological and infectious agents primarily via social and behavioral activities (e.g., sexually transmitted infections) are excluded. However, studies that included biological agents such as malaria and cryptosporidium are included by virtue of their exposure being directly associated with environmental conditions (i.e., vector breeding habitat and contaminated water). Program or project evaluations, reviews, and qualitative meta-syntheses of data from multiple studies are excluded.

Three areas of research that pertain to the field of environmental health but are not included in this analysis are briefly recognized. First, because this review focuses on proximate microlevel environmental hazards, studies that examine distal or macrolevel environmental concerns are excluded (i.e., in the domain of global change and environmental sustainability). Second, although public understanding of science is a field of research that provides data on how people translate or understand scientific information, and is important to environmental health scientists and risk communicators, it is not a study of environmental health per se, so such studies were excluded from the review. Third, studies of risk perception that examined the cognitive process of risk judgments that people make when they are asked to characterize and evaluate hazardous activities and technologies were excluded.

### Search strategy

A primary search included three terms: qualitative, environ*, and health [the asterisk (*) tells the search engine to include anything after that segment (e.g., environment, environs, environmental)]. The time frame of the search was from 1991 through 2008, beginning 2 years before the National Institute of Environmental Health Sciences (NIEHS) Environmental Justice Partnerships for Communications funding program, which encouraged multidisciplinary relationships among environmental health researchers. The primary search was conducted on ISI Web of Knowledge/Web of Science (Thomson Reuters, New York, NY), which includes social science citation indices and the National Library of Medicine’s MEDLINE database. More targeted searches included key-word and full-text searches in the electronic archives of the journal *EHP* and in the “Qualitative Research” collection of the *American Journal of Public Health* (*AJPH*), which dates back to 2000. These searches resulted in 3,155 records combined. Nearly 2,000 articles were immediately excluded because they were not qualitative papers and merely included the word “qualitative” in the text, or because they clearly met other exclusion criteria. The abstracts of approximately 1,160 articles were used to screen for papers that employed qualitative methods and met the definition of environmental health. If there was uncertainty, the paper was obtained and examined. The full texts of all papers included in this analysis were obtained, and the reference lists of these papers were examined for additional articles.

### Data extraction

The structure of this analysis borrows from literature on writing reviews (Badger et al. 2000; Higgins and Green 2006), quantitative meta-analyses (Petitti 2000), and qualitative meta-syntheses (Noyes and Popay 2007; Sandelowski et al. 1997). However, this is not a traditional review, meta-analysis, or meta-synthesis because not all the studies are on the same exposure or outcome (e.g., outcomes of the same clinical trial, or analyses of the same event or phenomenon), nor are the findings pooled and compared with a common metric.

As each article was identified for inclusion, it was read (or reread) and (re)considered for its ability to meet inclusion criteria. For every study, the following questions were asked: Is this a study about a physical, biological, or chemical exposure? Does this study discuss exposure in relation to health or perceived health risk? Does this study discuss health, or perceived health, in relation to exposure?

Once articles were included in the analysis, information relevant to the questions driving the analysis was extracted, entered into an Excel spread sheet, and further analyzed. Columns included journal title, authors, year of publication, environmental health topic (i.e., exposure and/or health outcome), qualitative methods, quantitative methods, analyses (of qualitative and quantitative data), findings and conclusions, author associations and disciplines, key words, context of study (if part of larger study or project), funding source, and country. Following the convention of meta-syntheses of qualitative studies, no studies are excluded for reasons of quality, nor is the quality of studies evaluated in this analysis (Sandelowski et al. 1997). Descriptions of qualitative research methods were extracted from each article, along with mentions of theoretical and analytical frameworks. Data for the findings and conclusion sections of all studies were initially extracted from the article abstracts so that the approach to data extraction would be as uniform as possible. When no abstracts were available, or when abstracts did not provide such information, the actual findings and conclusion sections of each article were examined for such data. Content of the spreadsheet was quantitatively summarized (e.g., number of articles published per year, number of articles written by each author, number of theories identified). These data were also examined for the frequency of specific exposures and outcomes. Qualitative content analysis was conducted to address each of the stated objectives. Content analysis refers to “any qualitative data reduction and sense-making effort that takes a volume of qualitative material and attempts to identify core consistencies and meanings” (Patton 2002). Specifically, the findings of articles were analyzed for themes, or frequently repeated ideas, in the types of information reported.

To aid the qualitative content analysis, the three columns of the Excel spreadsheet with large quantities of text (qualitative methods, analyses, and findings and conclusions) were imported as three separate documents into the qualitative analysis software NVIVO [version 7; QSR International (Americas), Cambridge, MA]. These data were then coded by the author. Codes are words and phrases used to tag units of data. Coding data, in this case article text, enables analysts to retrieve codes and associated data and to assign values of frequency, presence/absence, and relationship with other codes (MacQueen et al. 1998). Eventually these may be grouped under a theme that has been identified by the analyst(s). NVIVO preserved the table format of Excel so that coded data would not be disassociated with the authors of the study and year of publication (i.e., data retrieved by codes included the coded text and the identifying information). All data were coded with a total of 28 codes in the final code book I developed. Analysis was conducted twice at two distinct periods of time to achieve high intrarater reliability (Stemler 2001). Themes were identified in the data using the coverage and reference data provided by NVIVO, as well as consideration of a code’s meaning and its relationship with other codes. [See Supplemental Material, Table 1 (doi:10.1289/ehp.0901762) for a sample code book, and Supplemental Material, Figure 1 depicting the steps of qualitative analysis.]

## Data Synthesis

The following results were obtained from this analysis and grouped by the research objectives.

### Publishing qualitative environmental health research

Ninety-one articles met all inclusion criteria and were derived from 87 studies. The vast majority of articles (70 of 91) included multiple authors from three or more institutions or areas of discipline within a university. More than half of the articles included one or more authors from university departments within public health (e.g., environmental health, epidemiology, family and community medicine, health behavior and education, and health policy and management). Other health-related fields represented included clinical epidemiology, dermatology, health sciences, nursing, oncology, psychiatry, psychology, and tropical medicine. Areas of discipline outside of traditionally identified health fields included anthropology, geography, oceanography, urban and regional studies, and sociology. Eight included authors from government agencies and community-based organizations that participated in the research. Authors were from, and studies were conducted in Australia, Bangladesh, Belgium, Botswana, Brazil, Canada, Croatia, Cuba, Denmark, Finland, Germany, Ghana, Italy, Japan, Kenya, Nepal, the Netherlands, New Zealand, Nicaragua, Nigeria, Pakistan, Philippines, South Africa, Sweden, Syria, United States, and the United Kingdom and among native Australians and Alaskans. The most articles were written by authors in the United States, followed by the United Kingdom and then Canada.

Articles were published in 59 different journals (Table 1), with the most publications in a single journal totaling eight. Both journals with eight articles, *Social Science and Medicine* and *Health and Place*, are self-described interdisciplinary journals. This comparison among number of publication by journals does not take into consideration the frequency of publication for each journal or the relative size of each issue (number of articles published).

Three of the seven articles published by *EHP* are in mini-monographs (Furgal and Seguin 2006; Green et al. 2002; Lipscomb et al. 2005) and do not conform to the traditional format for a research publication in *EHP* (i.e., including a structured abstract and the traditional research article format of introduction, methods, results, and discussion). Three other *EHP* articles were published in the same supplement, titled “Migrant and Seasonal Farmworkers and Pesticides: Community-Based Approaches to Measuring Risks and Reducing Exposure” (Arcury and Quandt 2001). These articles also did not necessarily follow the research article format (Flocks et al. 2001; McCauley et al. 2001; Thompson et al. 2001). The seventh article in *EHP* (Thompson et al. 2008) was published as a traditional research article and reports further results of an intervention described in the aforementioned supplement. Similarly breaking from the usual journal format, two of the three articles in *AJPH* were published in the column “Public Health Matters” or “Framing Health Matters” (Héon-Klin et al. 2001; Wing et al. 2008) and not in the “Research and Practice” section of the journal, where papers reporting the results of research are typically published.

**Figure 1:**
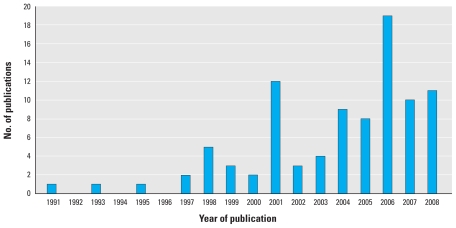
Number of articles published per year (1991–2008).

There appears to be an overall trend in the number of articles published per year (Figure 1), with noticeable spikes in 2001 and 2006. The one eligible article published in 1991 was funded by the Ontario Ministry of Environment with sponsorship by the Canadian Mental Health Association and was part of an ongoing “interdisciplinary research program to determine the impacts of exposure to environmental contaminants on human health and welfare and to develop strategies to reduce their adverse effects” (Taylor et al. 1991). Two of the three articles published in 1999 appear to be products of the same program funded in 1991, with the same funding source identified in the acknowledgments (Elliott et al. 1999; James and Eyles 1999).

### Methods and analysis reported in studies

This analysis identified a variety of qualitative techniques reported, with most studies relying on one-on-one interviews for their qualitative data. A subset of the studies also relied on quantitative techniques (i.e., mixed-methods qualitative and quantitative research). Approaches to data analysis were diverse, with details absent from a large number of studies.

#### Interviews

Most studies (65) used one-on-one interviews to collect data, with as few as six individuals interviewed in a study (Larsson et al. 2006) and as many as 93 (Messias and Lacy 2007). Approximately half of these studies also included data derived from other qualitative data collection techniques, such as focus groups (i.e., a group interview) and observation. Thirty-two articles reported focus groups, with numbers of groups per study ranging from one (Bush et al. 2001) to 32 (Amin and Basu 2004).

#### Observation

Sixteen studies included observation techniques. For example, a study of children’s vulnerability to water-related disease hazard in northern Pakistan conducted by a researcher from an American institution included observations with 30 households on details of household structure, household decision making, divisions of labor, child care, and recent illness events (Halvorson 2003).

#### Text analysis

Ten studies used document analysis as a data collection technique. Examples of sources included newspaper reports (Harper 2004), transcripts of congressional hearings on Gulf War illness (Shriver 2001), local print media and newsletters (Vandermoere 2006), e-mails (Imai et al. 2008), and the scientific literature in medical, public health, and epidemiologic journals (Brown et al. 2004; Wernham 2007).

#### Participatory research

Eleven articles described the use of participatory research methods, such that the authors’ research was designed or conducted in collaboration with the population being studied (Israel et al. 2006; Lambert et al. 2006; Wing et al. 2008). Three articles described their qualitative work as a component of community-based participatory research (Flocks et al. 2001; Lipscomb et al. 2005; McCauley et al. 2001). Neudoerffer et al. (2005) trained and hired members of the local community to be focus group facilitators and described participatory research as one method among many that are central in a vision of science for social change: “Methodological pluralism must be central to any new science for sustainability.” In their article, epidemiologic methods were complemented with qualitative tools described as participatory action research, Freirian conscientization, and appreciative inquiry. Several such approaches were described without elaboration, challenging the ability of a reader who is not familiar with this terminology to fully understand or appreciate the methods. Participatory rural appraisal was used to study climate change and vulnerability among Canadian northern Aboriginal communities (Furgal and Seguin 2006). Action research ethnography was conducted in an urban shantytown of Lagos, Nigeria, and was described as a qualitative strategy, “based on dialogical inquiries” (Jarvela and Rinne-Koistinen 2005). The better known techniques such as focus groups, in-depth interviews, and observation were also included in these studies.

#### Mixed qualitative and quantitative methods

Of the 91 articles, 35 included quantitative and qualitative data. More than half (18) of these mixed-methods studies were primarily epidemiologic studies that included a qualitative component. In four studies the only source of qualitative data came from open-ended (qualitative) questions on primarily closed-ended (quantitative) questionnaires (Doria et al. 2006; Elliott et al. 1999; Moffatt et al. 1995; Warr et al. 2007). More often, qualitative data from questionnaires was supplemented with data from observations and in-depth interviews. In several cases interviews followed up, or provided clarification on, survey data. On the other hand, preliminary interviews in at least two studies helped to construct and validate a larger, subsequent quantitative survey (Day 2006; Engvall et al. 2004). One study included in-depth interviews with 37 workers and used that information to construct a job exposure matrix (Lipscomb et al. 2005).

#### Theoretical frameworks and analyses

Despite differences in opinion about the extent to which specific theories should inform qualitative analysis, the contribution to theory was considered a defining characteristic of qualitative research. Theoretical frameworks have been called “the analyst’s reading glasses” and are conceptual models used in the process of interpretation (Malterud 2001). Roughly half (18) the environmental health research articles in this analysis made no reference to theoretical frameworks. Twenty-three articles included no description of how qualitative data were analyzed. Three of these were published in the journal *EHP* (Flocks et al. 2001; Lipscomb et al. 2005; McCauley et al. 2001), and none were traditionally structured research articles.

More than 30 articles described coding as a key component of analysis, with grounded theory being the most commonly cited analytic framework (Aragón et al. 2001; Bush et al. 2001; Green and Hart 1998; Hammal et al. 2005; Larsson et al. 2006; Schaefer-McDaniel 2007; Timmermans 2007; Trayers et al. 2006). Proponents of grounded theory research have suggested that analysts approach the data with no substantive theories in mind. All theories that emerge from the analyses are to be grounded entirely in the data, without preconceived notions of theories to which the findings may contribute. Grounded theory, which is strongly dependent on the process of coding, was developed by qualitative researchers who attempted to formalize their empirical methods when quantitative research was dominant in behavioral and social sciences (Snape and Spencer 2003).

Second to grounded theory was the mention of neighborhood effects. Although not referred to as an analytic framework per se, the theory of neighborhoods affecting health was explored in seven articles (Bowie et al. 2005; Israel et al. 2006; Michael et al. 2006; Schaefer-McDaniel 2007; Songsore and McGranahan 1998; Timmermans 2007; Warr et al. 2007). Other theoretical perspectives referred to in the analyses include ecological theory (Salazar et al. 2004; Schaefer-McDaniel 2007) and social constructionism (Moffatt and Pless-Mulloli 2003; Shriver 2001; Shriver et al. 1998). Two articles referred to the environmental stress and coping literature as an analytic framework (Haines et al. 2003; Wakefield et al. 2001).

### Qualitative contribution to environmental health sciences

In this analysis, all studies were analyzed from the perspective of an environmental epidemiologist, meaning that each study was examined for its contribution to environmental health understanding with respect to associations between a given environmental exposure and health outcome. Some studies naturally provided more information on one or the other, rather than measure a perceived or actual association between the two. Half a dozen studies were designed specifically to examine what might be considered effect measure modifiers (Amin and Basu 2004; Bickerstaff and Walker 2001; Day 2006) (e.g., socioeconomic status and sex). In what follows, the analysis of the studies’ findings and conclusions with respect to the types of information they contributed to the field of environmental health were organized under four headings: exposures, health outcomes, planning an intervention, and factors that influence environmental health. These were not mutually exclusive categories, and a single article may have been mentioned under all headings.

#### Exposures

**Figure 2:**
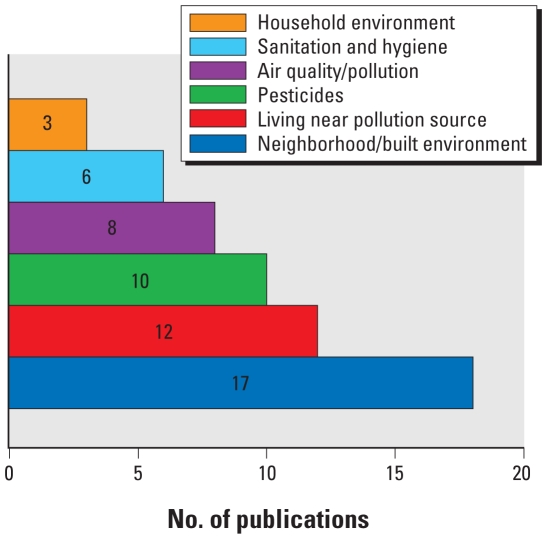
Environmental exposures studied in three or more publications.

Nearly all 91 studies provided data on environmental exposures. More than one-third of the studies focused on exposures known by scientists to be associated with health outcomes (e.g., lead in soil, the consumption of contaminated food or water, inhalation or dermal contact with pesticides, and air pollution) (Figure 2).

Most often, qualitative data identifies beliefs, activities, or behaviors that would increase exposure. For example, including children in focus groups about exposure to pesticides enabled the identification of a “large number of activities that may potentially expose children to pesticides through both direct and indirect routes” (Cooper et al. 2001). A dozen studies examined the perceived and actual exposures to residents living near pollution sources, including heavily industrial areas (Bush et al. 2001), solid waste facilities (Elliot 1998; Eyles et al. 1993; Taylor et al. 1991), confined area feed operations (Tu et al. 1997; Wing et al. 2008), mining operations (Moffatt and Pless-Mulloli 2003), and sites of a contamination event or disaster (Barnes et al. 2002; Messias and Lacy 2007; Shriver et al. 1998). Lambert et al. (2006) conducted a mixed-methods study using qualitative interviews, quantitative survey data, environmental sampling, and contaminant dispersion models to identify exposures in residential areas near the tar ponds of a coke and steel factory. Respondents in all areas of the study described the effects of ash deposition in the form of “dust,” “coal dust,” “dirt,” and “fall out” on and in their homes, cars, and laundry and in their community. According to Lambert et al. (2006), there were no differences in odors reported between the communities considered by authorities as adversely affected versus those considered to be free from contamination. Residents in areas supposedly free from contamination were reported to have provided researchers additional knowledge of child- specific exposures.

A relatively large number of studies examined aspects of the built environment or neighborhoods and possible associations with physical activity (Kamphuis et al. 2007; Krenichyn 2006; Michael et al. 2006; Regan et al. 2006; Richards and Smith 2007; Ries et al. 2008; Trayers et al. 2006; Yen et al. 2007). Most of these articles, and nine additional articles with a focus on neighborhood effects on health generally, were published since 2000 (Bolam et al. 2006; Bowie et al. 2005; Butchart et al. 2000; Day 2008; Israel et al. 2006; Popay et al. 2003; Schaefer-McDaniel 2007; Timmermans 2007; Warr et al. 2007).

A handful of studies identified new environmental exposures relevant to human health, or perceived to be hazardous by participants, that had not previously been considered by the study authors. Two such studies examined exposures of residents in urban renewal areas. In one, residents expressed concerns about potential risks due to gutting and demolition of buildings, in contrast to expressing positive reactions to the urban renewal that had been expected by urban planners (Bowie et al. 2005). A separate study reported that an element of a proposed redevelopment plan intended to improve health was perceived by residents to be a harbinger of crime: Focus groups revealed that residents were concerned that the proposed cycle/walkway would increase the vulnerability of their homes and cars to vandalism (Trayers et al. 2006). A third study that involved focus groups of African-American and Hispanic women in New York City identified a long list of environmental concerns among study participants that had not been anticipated by researchers, including needles, AIDS, drugs, violence, child abuse, domestic abuse, verbal and physical abuse, diseases, mental illness, pollution, rodents, broken-down buildings, and roaches (Green et al. 2002).

#### Health outcomes

Approximately one-third of the studies examined health outcomes. In most of these studies the health outcomes studied were previously suspected or known to researchers as generally being associated with environmental hazards (e.g., respiratory problems and air pollution, intestinal worms and hygienic practices). In some studies, however, health effects reported by participants represented new or previous undocumented outcomes. Focus groups and community workshops with Aboriginal communities in northern Canada associated respiratory stress among elderly participants with an increase in summer temperature extremes (Furgal and Seguin 2006). Participants described significant impacts of warming on ice travel and on hunting and fishing safety, with potential implications on food security and nutritional health. Furgal and Seguin (2006) wrote that there were anecdotal reports of “an increase in the number of accidents and drownings associated with poor or uncharacteristic ice conditions during times of the year that are predictable and typically very safe.”

In a study on the nonauditory health effects of aircraft noise exposure among children, Haines et al. (2003) noted that their results corroborated existing literature that noise annoyance is associated with feelings of mild irritation, anger, and fear. The authors were surprised to find that noise at home generated by neighbors created the highest annoyance among children: “Neighbor noise has been neglected in previous research of nonauditory health effects of noise exposure on children.” A quantitative survey may not have captured this unanticipated information.

**Figure 3:**
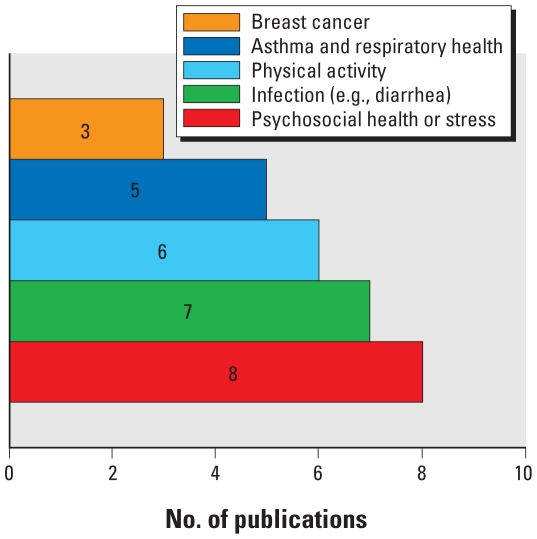
Health outcomes studied in three or more publications.

Environmental health research traditionally has not investigated associations between exposures to environmental hazards and mental and psychological health outcomes. However, a recurrent feature revealed in the analysis was the identification of psychosocial health effects directly and indirectly associated with environmental pollution. Psychosocial and stress-related health problems were the most frequently studied health outcomes among all the studies included in this literature analysis (Barnes et al. 2002; Bush et al. 2001; Elliott et al. 1999; Eyles et al. 1993; Haines et al. 2003; Israel et al. 2006; Moffatt et al. 1995; Vandermoere 2006) (Figure 3). Moffatt et al. (1995) conducted a study of anxiety and stress among residents living near a coking works; they found that residents’ suspicions that toxic emissions from the facility had damaged health were supported by the evidence of epidemiological studies on physical symptoms and health problems.

#### Planning an intervention

Several studies identified a target population for educational intervention based on the premise that people who are informed of health risks associated with exposure will act in ways to reduce or prevent exposure. A study on risk of water-related disease in northern Pakistan identified men as important targets for health education, given their influence and power over resource allocation in the home, where water-related hazards are most effectively controlled (Halvorson 2004). In a study conducted in Brazil, Peres et al. (2006) identified women as a target for pesticide education, because they can “significantly and unknowingly increase their exposure to these chemical agents and put their homes and families at risk, especially when the family’s regular clothes are washed together with contaminated ones.”

Most intervention-oriented studies revealed a complex set of social conditions that influence beliefs and behaviors that contribute to exposure to environmental health hazards. This is particularly true of studies conducted in developing areas regarding perceptions of the biological and chemical exposures associated with sanitation and hygiene (Aragón et al. 2001; Espino et al. 1997; Halvorson 2003, 2004; Hammal et al. 2005; Neudoerffer et al. 2005; Olsen et al. 2001; Peres et al. 2006; Phaswana-Mafuya and Shukla 2005) and with migrant farmworkers in the United States (Arcury et al. 2001, 2006; Flocks et al. 2001; McCauley et al. 2001; Salazar et al. 2004).

Power relations were a theme of several studies where failure to acknowledge official and unofficial power structures in the home, on the job, or in a community may have resulted in incomplete or inadequate intervention. Power structures included gender relations and disruption in such relations brought about by economic and demographic changes (Halvorson 2003) and cultural, economic, institutional, and psychosocial factors. For example, qualitative interviews with agricultural growers and extension agents participating in a particular study revealed the belief that the danger of agricultural pesticides and the incidence of pesticide-related poisoning had been greatly exaggerated by the general public, the media, and the government (Rao et al. 2004). The authors note that it was these people who were in positions of power to promote safe practices and enforce standards. These studies and their authors identified the importance of future interventions that consider the power structure that may exist in the relationship between those who are exposed and those who have the ability to mitigate such exposures.

#### Factors that influence environmental health

The identification of a broader context of risk perception was prominent among the 91 studies. To borrow from one study, lay concepts of health and illness generally included theories that “describe how the characteristics of particular areas combine with wider macro-structural factors to damage health via complex pathways including material, lifestyle and stress-related factors” (Popay et al. 2003). Such material, lifestyle, and sociopolitical factors were identified in many of the studies.

A focus group study of women in New York City that identified a huge variety of environmental health concerns among participants concluded: “Few programs address, much less reduce, the powerful social, political, and economic forces that push urban residents into ill health” (Green et al. 2002). This illustrates the findings of many studies that context-specific social, cultural, and economic circumstances shape perceptions of environment and health, and the relationship between the two (i.e., environmental health). This finding has been corroborated by more than a dozen studies that compared perceptions and responses of participants by differences in socioeconomic status, for example, by conducting focus groups and interviews with residents from the same city or neighborhood but from demographically distinct areas within the city or neighborhood (Bickerstaff and Walker 2001; Bolam et al. 2006; Bush et al. 2001; Day 2006, 2008; Israel et al. 2006; James and Eyles 1999; Kamphuis et al. 2007; Popay et al. 2003; Songsore and McGranahan 1998; Stevens et al. 2004; Timmermans 2007; Wakefield et al. 2001; Yen et al. 2007). Additionally, day-to-day experience of environmental hazards, or health conditions, was reported to influence perception of environmental health. Several studies reported that participants rely heavily on personal, tangible, experience of pollution over scientific evidence regarding the extent of exposures and health problems (Bickerstaff and Walker 2001; Day 2006; Olsen et al. 2001; Stevens et al. 2004; Wakefield et al. 2001).

The authors of several studies concluded that perceived ability to affect social and environmental change in one’s life affects how people perceive their environment and health (Bickerstaff and Walker 2001; Brown et al. 2004, 2006; Halvorson 2003; Harnish et al. 2006; Potts 2004). In his study of Gulf War illness, Shriver (2001) described senior officials in the Veterans Administration who reportedly controlled compensation claims and medical doctors working for the Veterans Administration as “social control agents.” According to Shriver’s analysis, they shaped public understanding of environmental illness and its diagnosis and treatment. Three studies identified the ability of lay activists to target and influence “control agents” (i.e., institutional, scientific, and social power structures) as a reason why the medical and scientific communities are now beginning to pay attention to possible environmental contributors to diseases. Potts (2004) compared the breast cancer and environment movements in the United States and in the United Kingdom and observed that in contrast with the United States, in the United Kingdom “women do not feel able to do anything about perceived hazards . . . translation of cynicism into action depends on knowledge and empowerment, which the United Kingdom movement has yet to mobilize.” Potts’s findings that activists in the United States have become empowered have been corroborated by another study of the breast cancer and environment movement that specifically identifies power sharing between scientists and lay people as a key component of calling attention to the possible environmental causation of breast cancer (Brown et al. 2006); as one activist interviewed said, “Power isn’t only knowledge. . . . It is bringing new ideas . . . to the table that scientists may not think about.”

### Limitations

For this analysis, several limitations should be noted. The articles discovered by search engines in the literature analysis do not represent the entire realm of qualitative environmental health research, as defined, published in peer-review journals. At least one specific article brought to the author’s attention would have met criteria but was not picked up in the search (Marko et al. 2004). Search engines are limited in their ability to identify and categorize such studies because of indexing practices of electronic databases. In particular, the titles and abstracts used to index qualitative studies often do not include summarized research methods (Evans 2002).

It is a limitation that the quality of studies was not evaluated in this analysis and that no attempt was made to examine articles for their mention of methods to reduce bias or to increase the validity of research results, how participants were identified and recruited, or the extent to which authors felt the results of their research might be generalized. Although worthy of consideration, these were not objectives of this analysis.

The literature analysis excluded books, book chapters, and “gray literature” (e.g., technical reports, working papers) that may be a substantial contribution to the field of environmental health.

## Discussion and Conclusions

Qualitative data are published in traditionally quantitative environmental health studies to a limited extent. However, this analysis demonstrates the potential of qualitative data to improve understanding of complex exposure pathways, including the influence of social factors on environmental health, and health outcomes. Qualitative data contribute to the understanding of population exposures by providing data on people’s behaviors, perceptions of risk, and the social, economic, cultural, and political considerations that influence personal exposure to environmental health hazards. In several studies these data would not have been captured using quantitative methods. This finding has consequences for the design of epidemiologic studies, particularly when this type of information may modify the relationship between exposure and illness (Lipscomb et al. 2005).

Incorporating qualitative methods into environmental health research may have implications for the types of exposures and outcomes typically studied by environmental health scientists. Many qualitative studies identified in this analysis address psychosocial health effects, including social stress, associated with environmental pollution. Some environmental health scientists are beginning to study the effects of physiological responses to stress on mechanisms that contribute to decreased cognitive function, abdominal obesity, hypertension, and other cardiovascular and immune diseases (Krieger 2001; Peterson 1999; Schulz and Northridge 2004). Biomedical approaches often do not incorporate mental and psychological processes. However, if stress is a psychological exposure that is differentially experienced by population subgroups, and the response to stress is physiological, then knowledge gained from qualitative and quantitative inquiry into the physical, sociocultural, and political processes that shape stress responses will further our understanding of the underlying causes and effects of physiologic responses to stress (Gee and Payne-Sturges 2004).

A large number of studies in this analysis focused on neighborhood effects on health. “Neighborhood” includes social, physical, biological, and chemical environment: where we live; what we live in; and the people, institutions, and social structures we live with. A number of quantitative epidemiological studies have identified moderate associations between neighborhood environment and mortality after adjusting for individual income, employment status, access to medical care, smoking, drinking, exercise, body mass index, and social ties (Kawachi and Berkman 2003). In addition to mortality risk, health outcomes associated with community context include low birth weight, asthma, injury, and cardiovascular disease (Sampson 2003). Studies identified in this analysis elucidate ways participants related health problems to the combined physical, psychological, and social environments in which they live. Macintyre and Ellaway (2003) suggested that many of the individual-level factors epidemiologists tend to control for (e.g., race, income, sex, education) represent variables on the causal pathway between neighborhood exposures and individual health outcomes that demand closer examination. For the qualitative researcher, these variables may represent a goldmine of potentially relevant information for understanding environmental health. Multilevel analyses of neighborhood- and individual-level characteristics and their contributions to stress suggest that such pathways should be further delineated (Schulz et al. 2008).

Results of this analysis confirm an observation made by Trostle (1986a) more than 20 years ago that most mixed-methods articles combining qualitative and quantitative methods look like the products of either social science or natural science. With few exceptions (Brown et al. 2006; Neudoerffer et al. 2005; Songsore and McGranahan 1998), the structure of articles more or less conforms to the style and norm appropriate to those intended for professionals in a particular discipline. From the perspective of an environmental health researcher familiar with the quantitative measurements of risk reported in public health journals, the findings of some social science articles included in the literature analysis were difficult to understand or summarize. The texts each speak to a particular kind of audience, each with a common discourse and shared professional jargon. An anthropologist or social scientist with a bias toward the development of theory might be surprised to find so few articles that provide details on analytic methods and theoretical frameworks. The techniques of qualitative research have been used for their practical application, but in few instances are findings related to theory. It is conceivable that theoretical considerations are a stumbling block for collaborations between qualitative and quantitative investigators, which might explain a relatively large number of publications focused on neighborhood effects on health—a theoretical framework that more than one discipline can hang its hat on. It is also possible that this analysis is a poor measure of actual collaboration between qualitative researchers and environmental health scientists and that intellectual cross-pollination is not well captured in results of such efforts that may be tailored for publication in one or another particular discipline.

No one journal could be identified as the intellectual home of qualitative environmental health research. Among all journals that published any qualitative environmental health research articles at all, the median of such articles per journal was one. It is possible that word limits set by publishers constrain the type of narrative often reported in qualitative studies; for example, the word limit is 8,000 for *Social Science and Medicine* articles, and 3,500 for the “Research and Practice” articles in the *AJPH*. The exceptions to the publishing format tend to prove the rule. Second to *Health and Place* and *Social Science and Medicine*, each with eight articles, the journal *EHP* published seven articles. These articles, however, were not typical *EHP* research articles; they reported on projects supported by the NIEHS community- based participatory research programs and did not conform to the traditional style of research article published in *EHP*. These NIEHS programs strongly encouraged and in some instances required the participation of lay people, or community residents, in community-based environmental health research. Such research programs, as described and supported by the NIEHS, sometimes included qualitative methods and may have contributed to its legitimatization in environmental health research (O’Fallon and Dearry 2002).

So far, qualitative techniques have not found a fixed home in environmental health scholarly literature. The use of qualitative data in the study of environmental health, however, does appear to be increasing over time, along with publications by interdisciplinary teams. Future work might examine opportunities for mixed-methods research, including the training of young environmental health investigators in the use of qualitative methods, and the development of recommendations for journals seeking to publish mixed-methods work such that analytic rigor would meet the standards of both quantitative and qualitative investigators.

## Figures and Tables

**Table 1:** Journals with environmental health studies using qualitative methods (1991–2008).

Journal	No. articles
*Acta Trop*	1
*Afr Health Sci*	1
*Afr J AIDS Res*	1
*AAOHN J*	1
*Am J Health Behav*	1
*Am J Health Promot*	2
*Am J Ind Med*	3
*Am J Public Health*	3
*BMC Public Health*	2
*Braz J Poult Sci*	1
*Chronic Dis Can*	1
*Crit Soc Pol*	1
*Ecohealth*	2
*Ecol Society*	1
*EcoSystem Health*	1
*Environ Health Perspect*	7
*Energy Policy*	1
*Environ Behav*	1
*Environ Urban*	1
*Global Environ Change*	1
*Health*	1
*Health Place*	8
*Health Risk Soc*	1
*Human Ecol Risk Assess*	1
*Hum Organ*	1
*Indoor Air*	1
*Inj Prev*	1
*Int J Health Serv*	1
*Int J Hyg Environ Health*	1
*Int J Occup Environ Health*	2
*Int J Urban Reg Res*	1
*J Adolesc Res*	1
*J Agric Saf Health*	1
*J Biosoc Sci*	1
*J Epidemiol Community Health*	2
*J Health Care Poor Underserved*	1
*J Nutr Educ Behav*	1
*J Public Health*	1
*J Urban Health*	2
*Malar J*	1
*Med Anthropol*	1
*Noise Health*	1
*Nurs Health Sci*	1
*Occup Med*	1
*Pediatr Pulmonol*	1
*Popul Stud (Camb)*	1
*Prof Geogr*	1
*Psychol Health*	1
*Public Health Nurs*	2
*Qual Health Res*	1
*Risk Anal*	1
*Sci Total Environ*	1
*Sociol Health Illn*	1
*Sociol Inq*	2
*Soc Sci Med*	8
*Sci Technol Human Values*	1
*Waste Manage Res*	1
*West J Nurs Res*	1
*World Dev*	1
Total	91
